# Synthesis, characterization, and *in vitro* and in silico antibacterial evaluation of 5-(3-nitrophenyl)-1,3,4-thiadiazol-2-amine

**DOI:** 10.3389/fchem.2026.1848149

**Published:** 2026-07-08

**Authors:** Abdelghani Sehmi, Farouk Boudou, Ahcene Keziz, Amal Belakredar, Huda Alsaeedi, Mikhael Bechelany, Gemma K. Kinsella, Ahmed Barhoum

**Affiliations:** 1 Department of Physical Science and Technology, Higher Normal School of Saida, Saïda, Algeria; 2 Department of Applied Molecular Genetics, Faculty of Natural and Life Sciences, University of Sciences and Technology of Oran, Mohamed-Boudiaf (USTO-MB), Oran, Algeria; 3 Department of Physics, and Chemistry of Materials Laboratory, University of M’sila, M’sila, Algeria; 4 Department of Biotechnology, Faculty of Natural Sciences and Life, University of Mostaganem Abdelhamid Ibn Badis, Mostaganem, Algeria; 5 Department of Chemistry, College of Science, King Saud University, Riyadh, Saudi Arabia; 6 Institut Européen des Membranes, IEM, UMR 5635, Univ Montpellier, ENSCM, CNRS, Montpellier, France; 7 School of Food Science and Environmental Health, Technological University Dublin, Dublin, Ireland; 8 School of Chemical and BioPharmaceutical Sciences, Technological University Dublin, Dublin, Ireland

**Keywords:** ADMET prediction, antibacterial activity, frontier molecular orbitals (FMO), green synthesis, molecular docking, molecular dynamics, thiadiazole

## Abstract

**Introduction:**

This study reports the synthesis, structural characterization, and antibacterial evaluation of 5-(3-nitrophenyl)-1,3,4-thiadiazol-2-amine (NPTA), a commercially available thiadiazole derivative with potential antimicrobial activity.

**Methods:**

NPTA was synthesized through a green, one-pot condensation reaction between thiosemicarbazide and 3-nitrobenzoic acid in absolute ethanol, affording a pale-yellow crystalline solid with a melting point of 110 °C–112 °C. The compound was characterized using Fourier-transform infrared (FTIR) and nuclear magnetic resonance (NMR) spectroscopy, confirming its structure. *In vitro* antibacterial assays, *in silico* ADMET and toxicity profiling, molecular docking, frontier molecular orbital (FMO) analysis, and 200 ns molecular dynamics simulations were performed.

**Results:**

*In vitro* antibacterial assays revealed significant activity against *Klebsiella pneumoniae* ATCC 13883,* Acinetobacter baumannii*, and *Listeria monocytogenes* ATCC 19114, with growth inhibition zones of 25.63 ± 0.17 mm, 29.09 ± 1.31 mm, and 26.65 ± 0.19 mm, respectively, and minimum inhibitory concentrations (MICs) ranging from 50 to 100 μg/mL. *In silico* ADMET and toxicity profiling predicted favourable drug-likeness, absorption, and safety. Molecular docking indicated strong binding affinities (−6.2 to −7.0 kcal/mol) with key bacterial targets, i.e., DNA gyrase subunit B (PDB: 1KZN) and penicillin-binding protein 4 (PDB: 3HUN). Frontier molecular orbital (FMO) analysis revealed a HOMO-LUMO energy gap of 3.79 eV, suggesting high electronic stability and reactivity. Furthermore, 200 ns molecular dynamics simulations confirmed the temporal stability of NPTA–protein complexes, particularly with DNA gyrase subunit B.

**Discussion:**

These results demonstrate the promising antibacterial potential of NPTA and support its further development as a multifunctional thiadiazole-based antimicrobial candidate.

## Graphical Abstract

**Figure:**
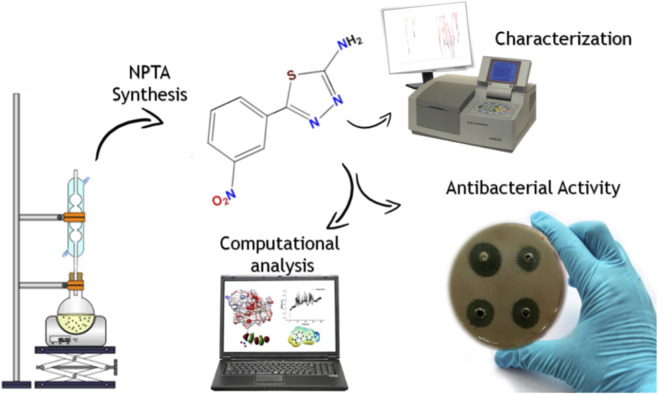

## Highlights

- NPTA was synthesized using a green, one-pot condensation approach.
- Structural characterization was confirmed by FTIR and ^1^H NMR spectroscopy.
- NPTA exhibited antibacterial activity against Gram-positive and Gram-negative bacteria.
- Molecular docking and molecular dynamics simulations showed stable protein–ligand interactions.
- FMO analysis indicated favorable electronic properties for antimicrobial activity.

## 1 Introduction

Antimicrobial resistance (AMR) is one of the most serious public health challenges worldwide (Shah et al., 2025). The World Health Organization has identified it as a major global health concern. According to recent reports, antimicrobial resistance contributes to a substantial burden of morbidity and mortality, with its impact expected to increase in the coming decades (Endale et al., 2023; Nazir et al., 2025). The widespread and inappropriate use of antimicrobial agents has reduced the effectiveness of many therapeutic options and complicated the management of infectious diseases (Owosagba et al., 2025). Bacteria can adapt to antimicrobial pressure through various mechanisms, including enzymatic drug inactivation, horizontal gene transfer, and target modification resulting from genetic mutations (Blair et al., 2015; Okesanya et al., 2025). These challenges highlight the importance of continuing efforts to discover and evaluate new antimicrobial compounds with diverse structural features and mechanisms of action that may complement existing therapeutic strategies (Pillaiyar et al., 2020).

Heterocyclic compounds are widely applied in antimicrobial drug discovery because they can interact effectively with essential microbial enzymes and display diverse pharmacological properties (Baranwal et al., 2023). Among them, 1,3,4-thiadiazoles have gained particular attention due to their favorable physicochemical characteristics, electronic properties, and structural flexibility (Addadi et al., 2025; Boudou et al., 2023). The electron-withdrawing nature of the thiadiazole ring supports strong binding interactions with bacterial targets, which can enhance antimicrobial activity (Boudjellal et al., 2025; Kumar et al., 2024). However, many thiadiazole derivatives reported in the literature exhibit narrow antimicrobial spectra or limited mechanistic investigation. In addition, several studies emphasize biological screening without integrating pharmacokinetic, toxicity, and molecular interaction analyses. Environmentally sustainable synthetic strategies for thiadiazole derivatives also remain insufficiently explored (Nainu et al., 2021; Tambe et al., 2021).

To address these limitations, the present study investigates a commercially available thiadiazole derivative, 5-(3-nitrophenyl)-1,3,4-thiadiazol-2-amine (NPTA), using an integrated experimental and computational approach. The introduction of a nitrophenyl substituent is expected to influence membrane permeability and interactions with microbial targets (Raimi et al., 2025). The reference strains selected for biological evaluation, *Klebsiella pneumoniae* ATCC 13883, *Acinetobacter* baumannii ATCC 17978, and *Listeria* monocytogenes ATCC 19114, are commonly used in antimicrobial screening studies and represent clinically relevant bacterial species (Hulankova, 2024). The novelty of this work lies in combining a green synthetic approach, comprehensive structural characterization, antibacterial evaluation, and *in silico* analyses to provide a broader understanding of the biological properties of NPTA. The findings contribute to the ongoing exploration of thiadiazole-based compounds as potential antimicrobial agents.

## 2 Experimental

### 2.1 NPTA synthesis

NPTA was prepared via a classical one-step synthesis, employing ethanol as a safe and environmentally friendly solvent. Briefly, 2 g of 3-nitrobenzoic acid (C_7_H_5_NO_4_, ≥98% purity, Sigma-Aldrich) and 1.16 g of thiosemicarbazide (CH_5_N_3_S, ≥98% purity, Sigma-Aldrich), 1:1 mole ratio, were combined with 30 mL of absolute ethanol (C_2_H_6_O, 99.8% purity, Sigma-Aldrich) in a 100 mL round bottom flask equipped with a reflux condenser and magnetic stirrer. Then, 1 mL of concentrated sulfuric acid (H_2_SO_4_, ≥ 98%, Sigma-Aldrich) was added dropwise under stirring over 5 min to initiate cyclization. Next, the reaction mixture was warmed in a steam bath at 90 °C–95 °C for 1 h and the reaction was monitored by thin layer chromatography (TLC) using n-hexane/ethyl acetate (9.5:0.5) as eluent on pre-coated silica gel F254 plates. After heating, the reaction mixture was left standing at room temperature for 1 h and then poured into a big beaker containing 100 g of broken ice. The suspension was left overnight (∼12 h) without disturbing it to allow full precipitation. The resulting yellow solid was vacuum filtered, washed in cold distilled water (3 × 10 mL), and recrystallized from 10 mL absolute ethanol through slow cooling. The yield (% w/w) was recorded, and the purity of the produced NPTA was confirmed by TLC and melting point determination (see Section 2.2). The obtained product was characterized using a variety of analytical techniques (Section 2.2).

### 2.2 NPTA chemical and structural characterization

The melting point was recorded using a Köfler hot-stage apparatus (Wagner & Munz) at the rate of 2 °C/min. The FT-IR spectrum was recorded on a JASCO 4000 spectrometer (Japan). To prepare the KBr pellet, 1 mg of compound was dissolved in 100 mg of anhydrous KBr and compressed under 10 tons pressure into a clear disc. Scans were acquired from 4000 to 500 cm^−1^ at a resolution of 4 cm^−1^. The ^1^H NMR spectrum was recorded on a Bruker Avance-400 spectrometer (399.875 MHz, Bruker) at room temperature. The solvent was deuterated DMSO (DMSO-d_6_) (0.5 mL, 99.9 atom % D, Sigma-Aldrich). Tetramethylsilane (Sigma-Aldrich) was used as internal standard (δ = 0 ppm). The sample concentration was ∼10,000 μg/mL, and 16 scans were summed to achieve the signal enhancement. TLC was used to assess the compound purity, employing UV light at 254 nm and iodine vapor staining for viewing. The Retention factor (R_f_) value was recorded.

### 2.3 *In vitro* antibacterial activity assessment by agar well diffusion

**FIGURE 1:**
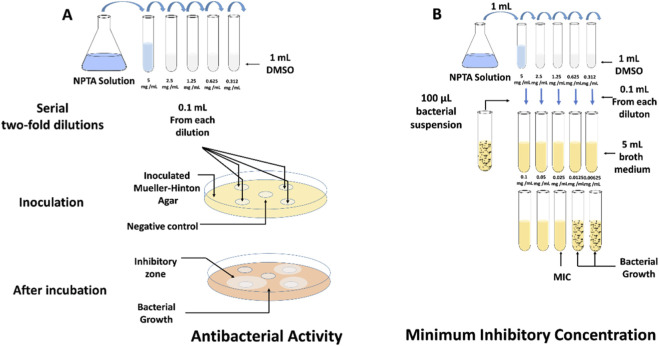
Schematic representation of the experimental procedures: **(A)** agar well diffusion assay used to evaluate the antibacterial activity of NPTA; **(B)** serial dilution method employed to determine the minimum inhibitory concentration (MIC).

The antibacterial activity of NPTA was investigated using *K. pneumoniae* ATCC 13883 (Gram-negative), *Acinetobacter baumannii* ATCC 17978 (Gram-negative) and *Listeria monocytogenes* ATCC 19114 (Gram-positive). These bacterial strains were chosen because of their clinical significance and multidrug resistance profiles, which are significant issues in present-day antimicrobial therapy (Nainu et al., 2021). Bacterial cultures were grown in tryptic soy broth at 37 °C for 24 h and diluted to a turbidity of 1.5 × 10^8^ CFU/mL using sterile 0.9% sodium chloride solution. The suspension concentration (CFU/mL) was confirmed by measuring the OD_600_ with a spectrophotometer. Sterile Müller-Hinton agar plates were prepared by pouring 20 mL of medium (autoclaved at 121 °C for 15 min) into each sterile Petri dish and allowing the solidification in aseptic conditions. Then, the bacterial suspensions were swabbed uniformly on the plate and left to dry in a biosafety cabinet for 10 min. Wells (6.0 ± 0.1 mm in diameter) were made using a sterile cork borer, and 50 µL of the compound (diluted to 5000, 2500, 1250, and 625 μg/mL in DMSO) was added in each well. DMSO alone (50 µL) was used as negative control. Gentamicin (50 µg/disc) and cefazolin (20 µg/disc) were used as positive controls (Raimi et al., 2025). Gentamicin and cefazolin were used as commercially available antibiotic discs (µg/disc; Cypress Diagnostics, Belgium), whereas NPTA was tested as a solution in the agar well diffusion assay (µg/mL). Due to the different experimental formats, direct conversion between µg/disc and µg/mL is not applicable. Plates were incubated at 37 °C for 24 h, and the diameter of the growth inhibition zones (in millimeters) was measured (Figure 1).

Plates were incubated at 37 °C for 24 h, and the diameter of the growth inhibition zones (in millimeters) was measured (Figure 1).

### 2.4 MIC determination using the broth dilution method

NPTA MIC was determined against selected bacterial strains using the standard broth microdilution assay (Hulankova, 2024). *K. pneumonia* ATCC 13883, *A*. *baumannii* ATCC 17978, and *L*. *monocytogenes* ATCC 19114 were cultured in Müller-Hinton Broth (MHB; Oxoid) at 37 °C overnight. The suspensions were adjusted to a turbidity of 1.5 × 10^8^ CFU/mL by OD_600_ measurement. All reagents and solutions were freshly prepared and filter-sterilized with 0.22 µm syringe filters. The stock solution of NPTA (1000 μg/mL) was prepared in DMSO (≥99.9% purity, Sigma-Aldrich) and stored at 4 °C and then diluted to 100, 50, 25, 12.5, 6.25, and 3.125 μg/mL in MHB (Sigma-Aldrich, USA, pH 7.3 ± 0.1 at 25 °C) using two-fold serial dilutions. All test tubes were filled with 4.9 mL of MHB and 100 µL of the corresponding NPTA dilution except the negative control (MHB alone) and positive control (MHB with DMSO and bacteria but without NPTA). To ensure that the final DMSO concentration (<1%) did not affect bacterial growth, an additional solvent control (MHB with DMSO alone) was added. Then, the standardized bacterial suspension (∼100 µL) was added into the tubes and shaken. After incubation at 37 °C for 18–24 h, turbidity was checked and the MIC was the lowest concentration of the compound that prevented visible growth. Experiments were repeated in triplicate (Figure 1).

### 2.5 Statistical analysis

NPTA antimicrobial activity was quantified in triplicate (n = 3) and data were presented as mean ± standard deviation (SD). Data were compared using SigmaPlot for Windows (version 11.0; Systat Software Inc., San Jose, CA, USA). Groups were compared with one-way analysis of variance (ANOVA) followed by the Tukey’s *post hoc* analysis. Significance was set at p < 0.05.

### 2.6 NPTA drug-likeness and toxicity assessment using *in silico* tools

NPTA drug-likeness, pharmacokinetic properties, and toxicity profiles were predicted *in silico* to evaluate its suitability as a drug candidate. SwissADME (http://www.swissadme.ch/index.php) was used to evaluate its absorption, distribution, metabolism and excretion (ADME), bioavailability, molecular properties, and medicinal chemistry friendliness, following established protocols (Hernandez et al., 2024). NPTA toxicological profile was estimated with the eMolTox server (http://www.emoltox.net), a program that predicts organ-specific and endpoint-related toxicities from the compound structure and functional groups (Ikhou et al., 2024). These *in silico* predictions provided a general description of NPTA drug-likeness, safety profile and bioactivity potential.

### 2.7 Quantum chemical and electronic structure calculations

The quantum chemical properties of NPTA were investigated using Density Functional Theory (DFT) in order to gain insight into its electronic structure and chemical reactivity. All calculations were carried out using the B3LYP hybrid functional in conjunction with the 6-311++G (d,p) basis set, including Grimme’s D3 dispersion correction with Becke–Johnson damping (GD3BJ), as implemented in the Gaussian 09W software package (Frisch et al., 2009). Gaussian 09, Revision D.01). GaussView was employed for molecular modeling, visualization of molecular orbitals, and charge distribution analysis.

The molecular geometry of NPTA was fully optimized, followed by vibrational frequency calculations to confirm the stability of the optimized structure. The energies of the Highest Occupied Molecular Orbital (HOMO) and the Lowest Unoccupied Molecular Orbital (LUMO) were determined, and the HOMO–LUMO energy gap (ΔEg was calculated to assess the chemical stability, global softness/hardness, and electronic transition characteristics of NPTA (Abid et al., 2023). In addition, the Molecular Electrostatic Potential (MEP) surface was generated to identify electron-rich (nucleophilic) and electron-deficient (electrophilic) regions, providing insight into potential sites for intermolecular interactions (Guan et al., 2025).

### 2.8 Molecular docking for predicting interactions with bacterial targets

Molecular docking studies were performed to investigate the potential interactions of NPTA with bacterial proteins involved in essential cellular processes. Two well-characterized antibacterial targets were selected from the Protein Data Bank (PDB): DNA gyrase subunit B from *Escherichia coli* (PDB ID: 1KZN) and penicillin-binding protein 4 (PBP4) from *Staphylococcus aureus* (PDB ID: 3HUN). DNA gyrase is an essential enzyme responsible for DNA replication, transcription, and chromosome maintenance, making it a validated target for several classes of antibacterial agents, including coumarin-based inhibitors such as clorobiocin. Likewise, PBP4 plays a crucial role in peptidoglycan biosynthesis and bacterial cell wall maintenance, which are indispensable for bacterial growth and survival. The selection of these proteins was therefore based on their established biological functions and their widespread relevance in antibacterial drug discovery.

**FIGURE 2:**
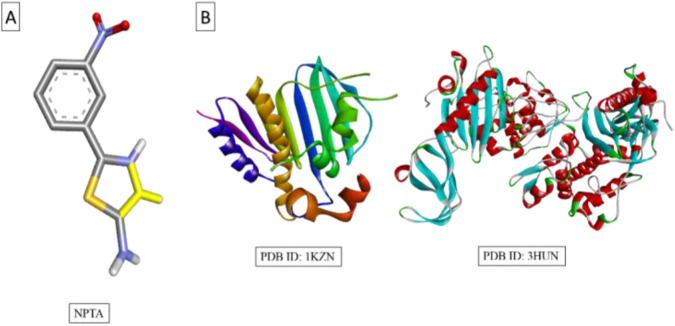
Three-dimensional crystal structures of the ligand NPTA **(A)** and the following target proteins **(B)** DNA gyrase subunit B (PDB ID: 1KZN), and *S. aureus* PBP4 (PDB ID: 3HUN). All structures were from the RCSB Protein Data Bank (www.rcsb.org).

The three-dimensional structure of NPTA (Figure 2A) was generated using MarvinSketch Version 5.10.0 (ChemAxon) and exported in PDB format. Ligand preparation and energy minimization were performed using AutoDock Tools 1.5.7, and the optimized structure was converted into PDBQT format for docking simulations. Protein structures were prepared by removing crystallographic water molecules, adding polar hydrogen atoms, and assigning Kollman charges before conversion into PDBQT files. All docking calculations were conducted using AutoDock Vina implemented in the PyRx Virtual Screening Tool (v0.8) (Trott and Olson, 2010).

The binding pockets of the target proteins were defined using the AutoGrid utility within PyRx, allowing the ligand to explore energetically favorable conformations within the active-site cavities. The grid box dimensions and center coordinates used for each target protein are summarized in Table 1.

**TABLE 1:** Grid box parameters for molecular docking simulations.

Target protein	Grid box size (Å) (X × Y × Z)	Centre coordinates (X, Y, Z)
PDB ID: 1KZN	25.647 × 30.136 × 30.979	19.080, 29.724, 34.611
PDB ID: 3HUN	41.714 × 42.136 × 40.888	−33.648, 13.630, −10.162

To validate the docking protocol and provide a meaningful context for interpreting the binding affinity of NPTA, the co-crystallized ligands clorobiocin (1KZN) and ampicillin (3HUN) were redocked into their respective binding sites under identical docking conditions. In addition, the experimentally tested antibacterial agents gentamicin and cefazolin were docked for comparative purposes. This strategy enabled comparison of NPTA with native ligands and reference antibacterial compounds, thereby facilitating a more objective evaluation of its predicted binding affinity and interaction profile. Docking scores, binding modes, and intermolecular interactions were subsequently analyzed to identify key residues involved in ligand recognition and stabilization within the active sites (Figure 2B).

The docking analyses were performed on a computer running Microsoft Windows 10 Professional equipped with an Intel® Core™ i3-7020U processor operating at 2.30 GHz and 4 GB RAM. The resulting protein–ligand complexes were visualized and analyzed using BIOVIA Discovery Studio Visualizer 2021 to examine binding orientations, intermolecular interactions, and docking energies.

### 2.9 MD simulations of protein–ligand complexes

To further confirm the docking results and assess the dynamic stability of the protein–ligand complexes, MD simulations were done using GROMACS 2023 with GPU support. The CHARMM36m force field was used to accurately describe biomolecular interactions in physiological conditions (Boudou et al., 2025; Kumar et al., 2024). Each docked complex was put into a triclinic simulation box and solvated using the TIP3P water model. Na^+^ and Cl^−^ ions were supplemented to neutralize the system and establish a physiological salt concentration of 150 mM. The system underwent energy minimization before the production run to eliminate steric clashes and achieve a low-energy state through two equilibration steps: (1) a fixed temperature and volume step (NVT ensemble), and (2) a fixed temperature and pressure step (NPT ensemble). Each step lasted 2 nanoseconds to allow the system to reach the thermal and pressure equilibrium. The response phase of the MD simulation was performed for 200 nanoseconds at 300° K and 1 bar pressure employing periodic boundary conditions and the Leap-Frog integrator. The Linear Constraint Solver (LINCS) algorithm was used to restrain all bond lengths. Long-range electrostatics were calculated with the Particle Mesh Ewald (PME) method. Trajectory analysis was performed after the simulation to evaluate the structural stability and interaction consistency. The structural parameters root-mean-square deviation (RMSD), root-mean-square fluctuation (RMSF) and radius of gyration (Rg) were calculated to monitor conformational changes, backbone flexibility, and the protein–ligand complex compactness over time. These parameters are standard metrics in molecular dynamics simulations, with RMSD values typically stabilizing below 2 Å, RMSF indicating residue-specific flexibility, and Rg values reflecting the compactness of the protein–ligand complex (Abouzied et al., 2024).

## 3 Results and discussion

### 3.1 NPTA synthesis using a single step cyclocondensation reaction (scheme 2)

**FIGURE 3:**
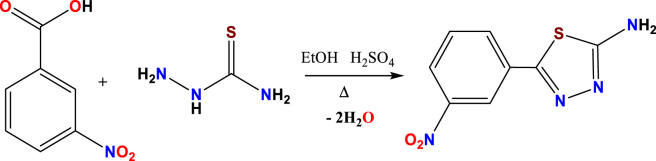
Synthetic route of 5-(3-nitrophenyl)-1,3,4-thiadiazol-2-amine.

Many reported methods for synthesizing thiadiazole derivatives suffer from drawbacks such as the use of strong Lewis acids, expensive reagents, toxic metal catalysts, and multistep procedures, as reported by Kumar et al. (2024) and Serban et al. (2018). To overcome these limitations, NPTA was synthesized via a simple one-step cyclocondensation reaction between thiosemicarbazide and 3-nitrobenzoic acid in absolute ethanol using concentrated H_2_SO_4_ as a dehydrating and cyclizing agent (Figure 3). The reaction proceeds through protonation of the carboxyl group, followed by nucleophilic attack of the terminal–NH_2_ group of thiosemicarbazide, leading to thiadiazole ring formation.

This provides a critical amide intermediate that is cyclized intramolecularly through substitution and elimination reactions to yield the 1,3,4-thiadiazole ring. Aromatization and stabilization of the thiadiazole nucleus are brought about by H_2_SO_4_-catalyzed oxidative dehydrogenation. The obtained NPTA was a pale-yellow crystalline solid upon cooling and was purified by recrystallization using ethanol. TLC analysis yielded a retention factor of 0.42 and the compound exhibited a melting point of 110 °C–112 °C, indicating that it was highly pure, in agreement with the literature on thiadiazole analogs (Serban et al., 2018). The synthetic route was excellent concerning atom economy and ease of operation without the need of numerous purification steps and toxic reagents. The conversion efficiency was extremely high as indicated by the reaction yield of 56% (1.48 g). Moreover, it is a green synthesis process because it uses ethanol, a comparatively non-toxic solvent, and one-step processing with minimal waste and reaction time. Compared with other thiadiazole synthesis methods, which are often multi-step or require the use of coupling agents, this single-step reaction provides a low-cost and reproducible pathway with good stoichiometric balance and reaction rates.

### 3.2 NPTA structure confirmation by FT-IR and ^1^H NMR spectroscopy

**FIGURE 4:**
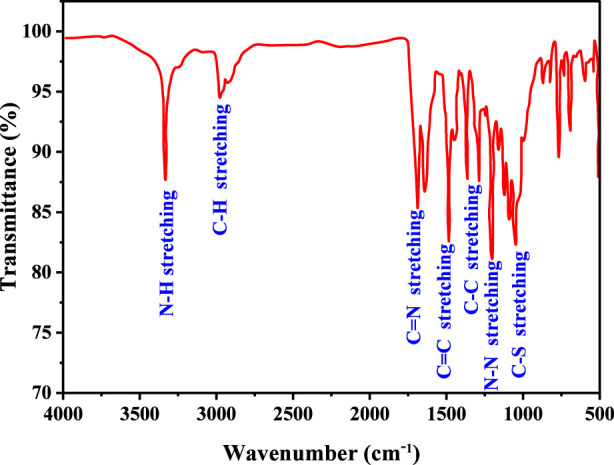
FT-IR spectrum of 5-(3-nitrophenyl)-1,3,4-àthiadiazol-2-amine (NPTA).

NPTA structure was confirmed by FT-IR and ^1^H NMR spectroscopy. The compound structure was determined from the FT-IR spectrum (Figure 4). N–H stretching vibrations were responsible for the strong band at 3339 cm^−1^. Aromatic C–H stretching was indicated by the band at 2985 cm^-1^. The strong band at 1696 cm^−1^ was assigned to C=N stretching in the thiadiazole ring, confirming the heterocyclic core formation. The band at 1488 cm^−1^ was due to C=C stretching in the phenyl ring. The band observed at 1362 cm^−1^ was assigned to C–C stretching, confirming the aromatic structure. The bands located at 1204 cm^−1^ and 1044 cm^−1^ were attributed to the N-N and C–S stretching vibrations of the thiadiazole ring, respectively, in agreement with previously reported data (Mohamed et al., 2025).

**FIGURE 5:**
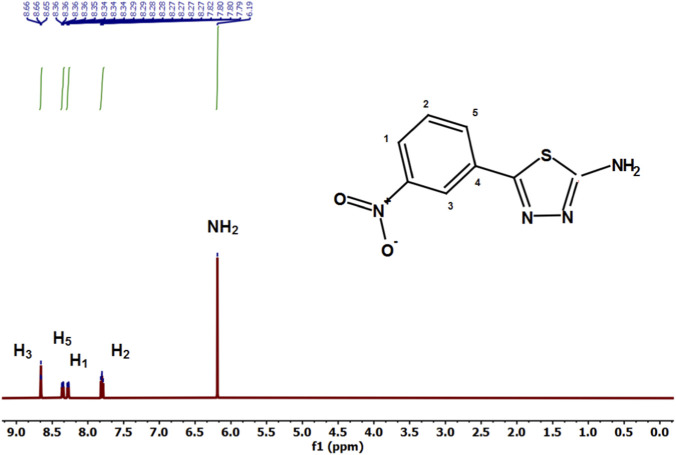
^1^H NMR spectrum of 5-(3-nitrophenyl)-1,3,4-thiadiazol-2-amine (NPTA).

The ^1^H NMR spectrum was recorded in DMSO at 400 MHz. It showed well-separated signals at δ 8.66 (t, J = 2.2 Hz, 1H; meta C-H functionalization of the nitro group), δ 8.35 (ddd, J = 8.4, 2.1, 1.1 Hz, 1H; assigned to C3-H), δ 8.28 (ddd, J = 7.9, 2.2, 1.2 Hz, 1H; assigned to C1-H), δ 7.80 (dd, J = 8.5, 7.8 Hz, 1H; assigned to C6-H), and δ 6.19 (singlet, 2H), corresponding to the nitrophenyl aromatic protons and the amine protons of the thiadiazole ring (Figure 5). These chemical shifts and coupling constants agree with the proton environments of the proposed molecular structure, confirming the electronic distribution and substitution pattern of the compound. The two-proton singlet at δ 6.19 ppm confirmed the presence of the primary amine (–NH_2_) functional group at position 2 of the thiadiazole ring. These spectroscopic findings are strong evidence of the successful synthesis and stability of NPTA and confirmed the anticipated chemical structure.

### 3.3 NPTA antibacterial activity

**FIGURE 6:**
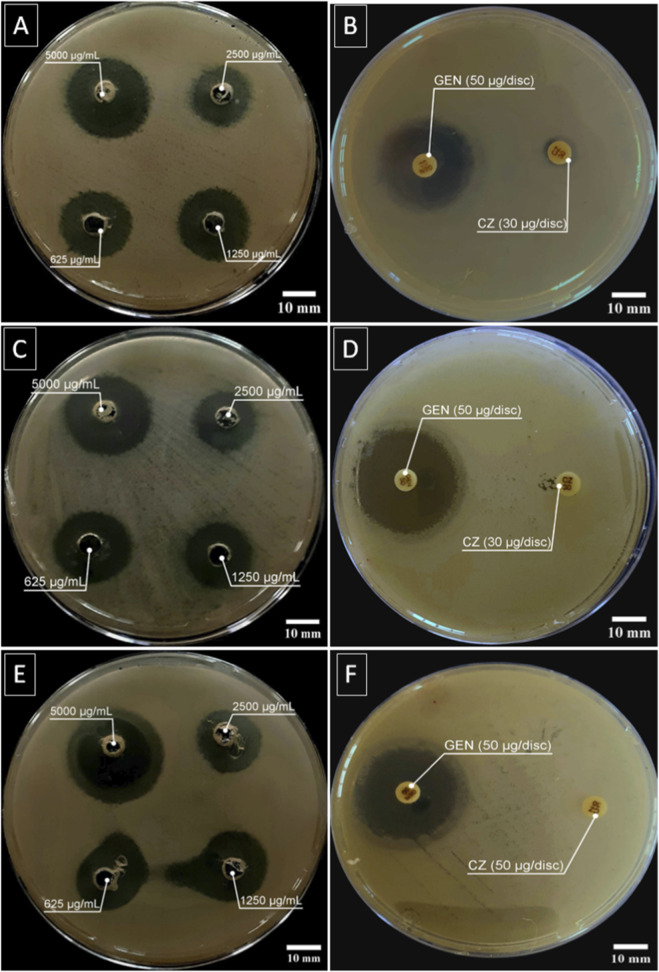
Agar well diffusion assay showing the antibacterial activity of NPTA at the indicated concentrations against: **(A)** *K*. *pneumoniae* (ATCC 13883), **(C)** *L*. *monocytogenes* (ATCC 19114) and **(E)** *A*. *baumannii* (ATCC 17978) and of gentamicin (GEN; 50 µg/disc) and cefazolin (CZ; 20 µg/disc), positive controls, against **(B)** *K. pneumoniae* (ATCC 13883), **(D)** *L. monocytogenes* (ATCC 19114) and **(F)** *A. baumannii* (ATCC 17978) in the same experimental conditions.

The antibacterial potential of NPTA was tested using a set of bacterial pathogens and the agar well diffusion and broth macrodilution methods. NPTA was broadly active against all tested pathogens in a dose-dependent manner. In the agar well diffusion test (Table 2; Figure 6), NPTA was highly active at the maximum concentration tested (5000 μg/mL) against *A*. *baumannii* ATCC 17978 (growth inhibition zone diameter = 29.09 ± 1.31 mm), followed by *L*. *monocytogenes* ATCC 19114 (growth inhibition zone diameter = 26.65 ± 0.19 mm) and *K*. *pneumonia* ATCC 13883 (growth inhibition zone diameter = 25.63 ± 0.17 mm). These results indicate that NPTA is very active against Gram-negative and Gram-positive bacteria. This spectrum agrees with reports that thiadiazole derivatives act as DNA-gyrase inhibitors and display potent activity against *Acinetobacter baumannii* and *Pseudomonas aeruginosa* (Cotman et al., 2023). For comparison, the control antibiotic gentamicin (50 µg/disc) gave larger growth inhibition zone diameters for all tested strains, particularly against *A. baumannii* (32.52 ± 1.89 mm). Conversely, cefazolin (20 µg/disc) inhibited only *K. pneumoniae* ATCC 13883 and very slightly (10.26 ± 0.14 mm). This indicates a relatively better efficacy of NPTA compared with cefazolin.

**TABLE 2:** Growth inhibition zone diameters (mm) of NPTA and standard antibiotics against tested bacterial strains.

Tested antimicrobial agents	Concentration	Unit	Volume per well (µL)	*K. pneumoniae ATCC 13883*	*L. monocytogenes ATCC 19114*	*A. baumannii ATCC 17978*
NPTA	5000	µg/mL	50	25.63 ± 0.17	26.65 ± 0.19	29.09 ± 1.31
NPTA	2500	µg/mL	50	19.29 ± 0.53	19.09 ± 0.12	19.40 ± 0.46
NPTA	1250	µg/mL	50	22.01 ± 0.10	20.10 ± 0.69	21.75 ± 0.18
NPTA	625	µg/mL	50	22.49 ± 1.01	22.21 ± 0.66	20.64 ± 2.58
Gentamicin (disc)	50	µg/disc	—	29.02 ± 0.11	32.46 ± 1.28	32.52 ± 1.89
Cefazolin (disc)	20	µg/disc	—	10.26 ± 0.14	ND	ND

Gentamicin and cefazolin were used as commercial antibiotic discs (µg/disc; Cypress Diagnostics, Belgium), whereas NPTA was tested as a solution (µg/mL).

**FIGURE 7:**
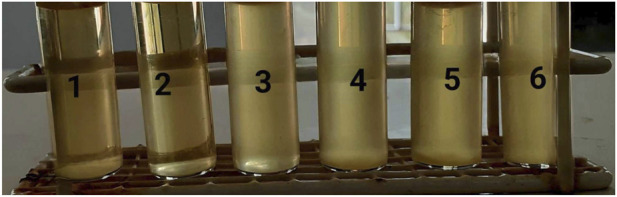
Minimum Inhibitory Concentration (MIC) evaluation showing inhibition of bacterial growth in tubes 1 and 2 and growth (cloudy) in tubes 3 to 6.

The MIC assay (Figure 7) further confirmed the antibacterial activity of NPTA. The compound inhibited the growth of *K. pneumoniae* ATCC 13883 and *A. baumannii* ATCC 17978 at a minimum concentration of 100 μg/mL, whereas complete inhibition of *L. monocytogenes* ATCC 19114 was observed at 50 μg/mL. In contrast, the tubes containing lower NPTA concentrations (25, 12.5, 6.25, and 3.125 μg/mL) exhibited visible turbidity, indicating bacterial growth. These MIC values (50–100 μg/mL) are consistent with previous reports demonstrating the antibacterial potential of thiadiazole derivatives against both Gram-positive and Gram-negative bacteria, possibly through inhibition of DNA gyrase and other essential bacterial targets (Yılmaz et al., 2025). Overall, the MIC findings corroborate the agar well diffusion results, confirming the concentration-dependent antibacterial activity of NPTA, with *L. monocytogenes* ATCC 19114 being the most susceptible strain.

### 3.4 In silico prediction of NPTA drug-likeness and toxicity profiles

**FIGURE 8:**
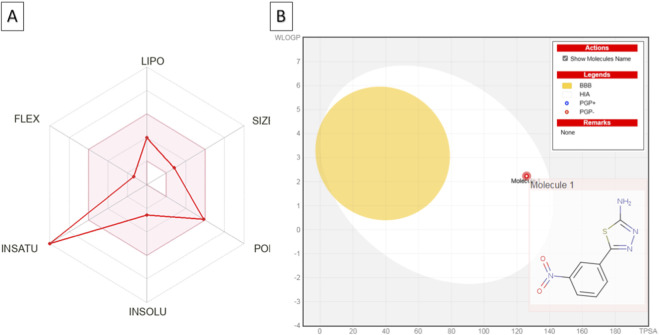
SwissADME analysis of NPTA. **(A)** Bioavailability radar plot showing optimal lipophilicity, suitable polarity and acceptable molecular flexibility, minimal insolubility and moderate unsaturation. The colored zone indicates the optimal range for each property and the red line the actual range. **(B)** BOILED-Egg plot showing the blood brain barrier absorption potential (yellow). The red dot indicates that NPTA should not be a p-glycoprotein substrate. LIPO: lipophilicity, POLAR: polarity, INSOLU: insolubility, INSATU: unsaturation, FLEX: flexibility, BBB: blood-brain barrier, HIA: human intestinal absorption, PGP+: p-glycoprotein substrate, and PGP-: not p-glycoprotein substrate.

**FIGURE 9:**
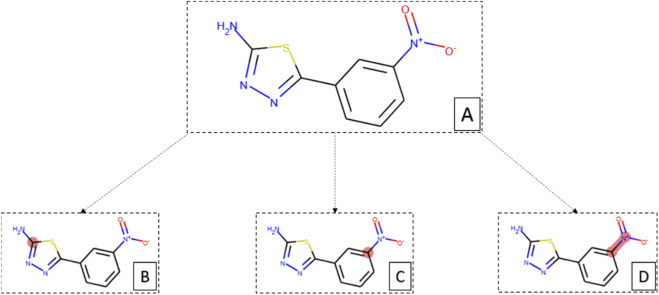
Predicted potential toxicity of NPTA and its possible toxic substructures (highlighted in red). **(A)** Representation of the molecule. **(B)** Idiosyncratic toxicity via metabolic activation. **(C,D)** Covalent interactions with DNA.

NPTA drug-likeness was evaluated using *in silico* approaches to predict its oral bioavailability and toxicity. The radar plot obtained with the SwissADME tool indicated that NPTA exhibited favorable physicochemical properties, including optimal lipophilicity, suitable polarity and acceptable molecular flexibility, with minimal insolubility and moderate unsaturation (Figure 8A). Quantitatively, SwissADME (https://www.swissadme.ch) predicts that NPTA is soluble in aqueous media, giving estimated solubilities of 0.585 mg mL^−1^ (2.63 × 10^−3^ M, ESOL model), 0.0385 mg mL^−1^ (1.73 × 10^−4^ M, Ali model) and 0.251 mg mL^−1^ (1.13 × 10^−3^ M, SILICOS-IT model); the compound remained stable in all biological assays at micromolar to low-millimolar concentrations. Such properties are in line with the characteristics commonly associated with orally bioavailable compounds, suggesting that NPTA should display optimal absorption and distribution in biological systems. These findings are consistent with those reported by Azzam (2023), who demonstrated that compounds with similar physicochemical profiles exhibited favorable oral bioavailability. Moreover, the BOILED-Egg plot indicated that NPTA falls within the desired region for gastrointestinal absorption, which is favorable for its oral bioavailability potential (Figure 8B), and is not a p-glycoprotein substrate. This suggests that it may evade active efflux from the central nervous system. This property is useful for maintaining the therapeutic concentration in the central nervous system and could be particularly suitable for the management of brain infections. The eMolTox tool was used to predict NPTA toxicity. The structure of NPTA adhered to drug-like frameworks; however, there were few potential safety concerns (Figure 9). Specifically, this tool predicted idiosyncratic toxicity via metabolic activation of the compound to reactive intermediates that could initiate unpredictable toxicities (Figure 9B) as well as potential covalent interactions with DNA (Figures 9C,D), with a genotoxic risk. These interactions have the potential to alter key cellular processes by interfering with DNA replication and transcription. These findings are in agreement with Ghosh et al. (2021), who applied *in silico* approaches to predict potential toxicity of antiviral compounds.

### 3.5 Frontier molecular orbital (FMO) and reactivity analysis

**FIGURE 10:**
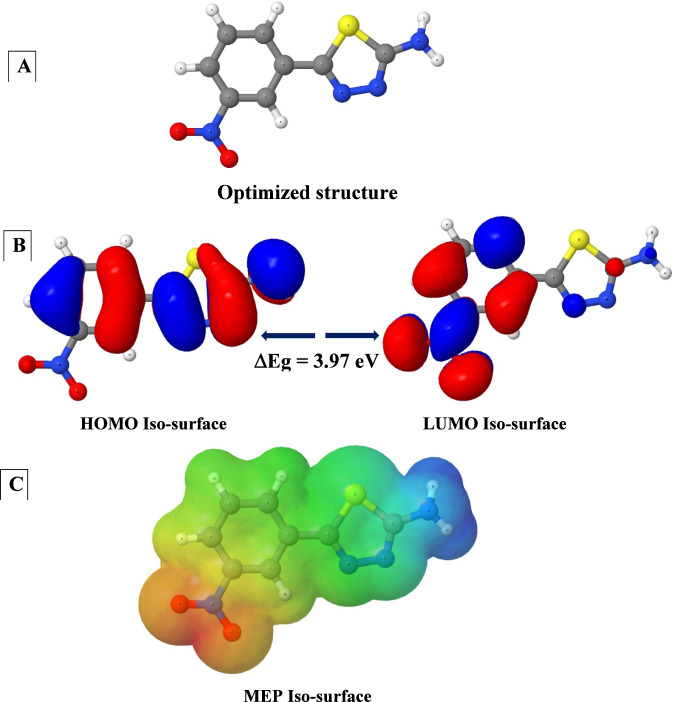
Frontier molecular orbital analysis of NPTA showing the optimized molecular structure **(A)**, frontier molecular orbitals **(B)**, and electrostatic potential analysis results **(C)**.

NPTA molecular geometry was optimized to find its FMO energies (Figure 10A). From the calculation, the HOMO energy was −2.92 eV and the LUMO energy was −6.71 eV, which gives an energy gap (ΔEg = E_LUMO_ − E_HOMO_) of 3.79 eV (Figure 10B). A low band gap is considered an indicator of both electronic polarizability and high reactivity, suggesting that NPTA can be readily involved in charge transfer reactions. The HOMO–LUMO gap also provided some insights into NPTA chemical stability and reactivity. Indeed, smaller gaps are features of molecules with higher chemical softness and susceptibility to electron transfer reactions. The spatial patterns for the HOMO and LUMO orbitals (depicted in Figure 10B) also provided different localization patterns. The HOMO orbital was predominantly localized throughout the electron-rich π-conjugated system, suggesting possible sites for electron donation. The LUMO orbital extended across the electron-deficient regions, suggesting favorable sites for electron acceptance. These observations are consistent with the findings of Ebenezer et al. (2022) (Ebenezer et al., 2022), who reported similar HOMO and LUMO distributions in nitrogen-containing heterocyclic medicinal compounds.

Then, the MEP surface was mapped to assess NPTA reactivity (Figure 10C) and obtain a visual representation of the electron distribution and electrostatic potential on the molecular surface. The MEP analysis showed that oxygen atoms of the nitro group (−NO_2_) were susceptible to attack by electrophilic attack due to the high electron density (red regions). Nitrogen atoms of the thiadiazole ring also exhibited partial negative charge under mesomeric and inductive effects, reflecting greater chemical reactivity. Conversely, the amine group (−NH_2_) displayed relatively positive potential (blue), indicating that it is susceptible to nucleophilic attack. Moreover, the sulfur atom of the thiadiazole ring was mapped in the yellow-shaded intermediate region, suggesting modest electrostatic activity and the probable involvement in intermolecular interactions, such as hydrogen bonding [41]. The charge distribution pattern in the MEP map reflects a well-balanced electronic architecture that might allow interacting with various biological targets. The presence of highly polar regions around the NO_2_ and NH_2_ groups also favors hydrogen bonding, which could explain NPTA antimicrobial activity. Altogether, the HOMO–LUMO analysis and MEP mapping confirmed that NPTA has a reactive electronic structure with well-defined electrophilic and nucleophilic regions of interaction, strengthening its position as a biologically active and multifaceted molecule (Azzam, 2023).

In the MEP map (Figure 10C), the red to yellow regions indicates areas of high electron density (negative potential) typically attractive to electrophiles. The blue regions indicate areas of electron deficiency (positive potential) where nucleophilic attack is favoured. The green regions are neutral electrostatic regions with low reactivity.

### 3.6 Molecular docking and binding interaction analysis

**FIGURE 11:**
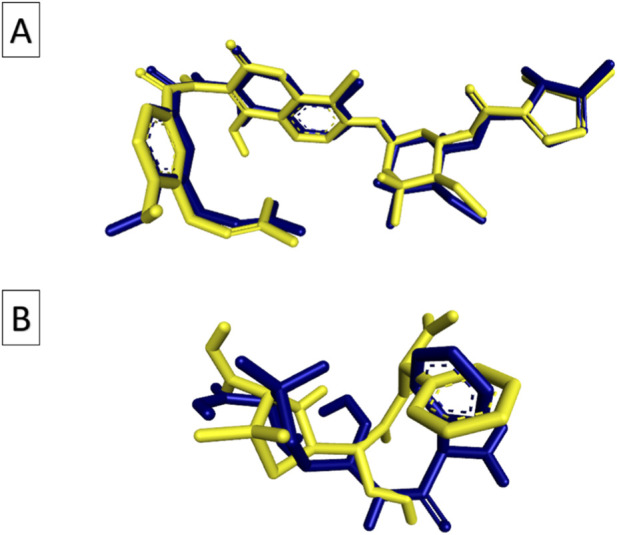
Validation of the docking protocol by redocking of native ligands into the active sites of target proteins. **(A)** 1KZN with Chlorobiocin showing a binding energy of −8.83 kcal/mol and a calculated RMSD of 0.217 Å, and **(B)** 3HUN with Ampicillin showing a binding energy of −7.78 kcal/mol and a calculated RMSD of 1.357 Å. Native ligands are shown in blue and redocked ligands in yellow.

The docking protocol was validated through redocking of the native ligands into the binding sites of the target proteins. The results showed very low root mean square deviation (RMSD). For the 1KZN complex, the best redocked pose exhibited a binding energy of −8.828 kcal/mol with a calculated RMSD of 0.217 Å. Similarly, for the 3HUN complex, the best pose showed a binding energy of −7.78 kcal/mol and a calculated RMSD of 1.357 Å. These low RMSD values indicate a strong agreement between the experimental and predicted binding poses, confirming the accuracy and reliability of the docking protocol in reproducing the native ligand orientations within the active sites (Figure 11).

To contextualize the binding affinity of NPTA, the co-crystallized ligands and experimental controls were docked into the same binding pockets under identical conditions (Table 3). Chlorobiocin (the native ligand of 1KZN) exhibited a binding energy of −8.83 kcal/mol, while NPTA scored −6.8 kcal/mol, indicating that NPTA possesses favorable but weaker binding than the established coumarin inhibitor. Ampicillin (the native ligand of 3HUN) scored −7.78 kcal/mol compared to NPTA’s −7.0 kcal/mol, suggesting that NPTA approaches but does not exceed the binding strength of the co-crystallized β-lactam. Critically, gentamicin—an aminoglycoside antibiotic that primarily targets the 30S ribosomal subunit and not DNA gyrase or PBPs—showed poor docking scores in both sites (−4.2 kcal/mol for 1KZN and −4.5 kcal/mol for 3HUN), confirming that NPTA engages these targets through a distinct mechanism from aminoglycosides. Cefazolin, a β-lactam antibiotic that targets PBPs, scored −6.9 kcal/mol in 3HUN but only −4.8 kcal/mol in 1KZN, consistent with its known mechanism of action.

**TABLE 3:** Comparative binding affinity and molecular interactions of target proteins with NPTA, co-crystallized ligands, and control antibiotics.

Compound	Target protein (PDB ID)	Estimated free energy of binding (kcal/mol)	Residues involved in bonded interactions	Interaction modes
NPTA	DNA gyrase subunit B (1KZN)	−6.8	ASP A:73, ALA A:47, VAL A:71, THR A:165, ASN A:46, ILE A:78	Hydrogen bond, π-σ, amide-π stacked, π-alkyl
NPTA	PBP4 from *S. aureus* (3HUN)	−7.0	LYS A:259, THR A:260, SER A:139, SER A:116, GLU A:114	Hydrogen bond
Chlorobiocin (co-crystallized)	DNA gyrase subunit B (1KZN)	−8.83	ASP A:73, GLY A:77, ASN A:46, ARG A:136, ILE A:78	Hydrogen bond, π-π, π-alkyl, van der waals
Ampicillin (co-crystallized)	PBP4 from *S. aureus* (3HUN)	−7.78	LYS A:259, THR A:260, SER A:139, ASN A:161	Hydrogen bond, π-alkyl, salt bridge
Gentamicin (control)	DNA gyrase subunit B (1KZN)	−4.2	THR A:165, ASN A:46	Hydrogen bond (weak)
Gentamicin (control)	PBP4 from *S. aureus* (3HUN)	−4.5	SER A:116, GLU A:114	Hydrogen bond (weak)
Cefazolin (control)	DNA gyrase subunit B (1KZN)	−4.8	ASP A:73, ALA A:47	Hydrogen bond (weak)
Cefazolin (control)	PBP4 from *S. aureus* (3HUN)	−6.9	LYS A:259, THR A:260, SER A:139	Hydrogen bond, π-alkyl

These comparative data demonstrate that NPTA’s binding energies are substantially more favorable than those of mechanistically inappropriate controls and approach those of native ligands, supporting the biological relevance of the docking predictions. The binding affinities of NPTA, estimated by calculating the free energy of binding (ΔG), were −6.8 kcal/mol for 1KZN and −7.0 kcal/mol for 3HUN. Whereas stronger interactions were observed with 3HUN, reflecting molecular complementarity and differences in interaction dynamics in the active sites.

Protein–ligand complexes interact with amino acid residues through hydrogen bonds, π-interactions, and van der Waals contacts. For example, the 1KZN-NPTA complex interacted through hydrogen bonds and π-σ interactions with amino acid residues such as ASP A:73 and ALA A:47, and the 3HUN-NPTA complex mostly through hydrogen bonds with LYS A:259 and THR A:260. These observations demonstrate the multi-target interaction profiles of NPTA with different bacterial proteins.

**FIGURE 12:**
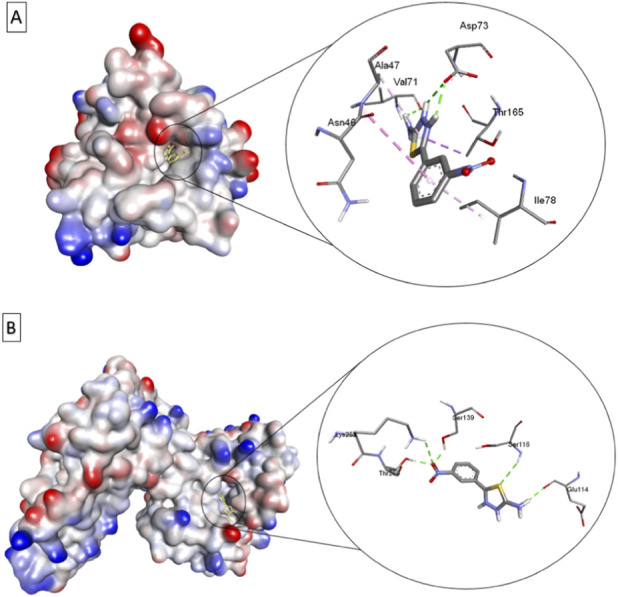
Surface views of the target protein-NPTA (ligand) complexes (left), enzyme active site, and 3D structure of the target protein-NPTA bonds (right). The complexes depicted are: **(A)** DNA gyrase subunit B (1KZN)-NPTA, and **(B)** *S. aureus* PBP4 (3HUN)-NPTA.

**FIGURE 13:**
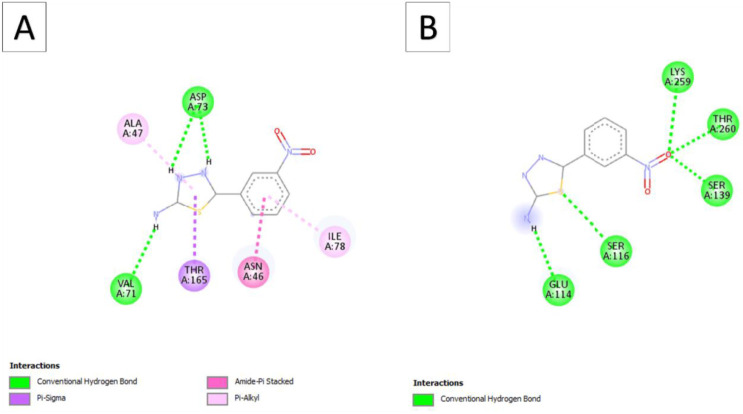
2D visualizations of the interaction modes between NPTA and target proteins. **(A)** DNA gyrase subunit B (1KZN)-NPTA complex, and **(B)** *S. aureus* PBP4 (3HUN)-NPTA complex.

Figure 12 illustrates the surface topography of each protein–ligand complex, the binding pocket accessibility, and NPTA spatial complementarity in the enzyme active sites. The three-dimensional (3D) insets and overlays give the exact interactions, such as hydrogen bonding and hydrophobic contacts, that explain complex stability. Figure 13 complements these results with two-dimensional (2D) interaction diagrams that clearly mark hydrogen bonds, π-interactions, and interacting residues. The 2D maps provide quick references to demonstrate key binding residues and assess interaction mechanisms.

The molecular docking results collectively indicate that NPTA forms energetically favorable and structurally consistent interactions with the tested bacterial target proteins, with binding affinities that are contextually supported by comparison with co-crystallized ligands and mechanistically relevant controls. These findings suggest that NPTA may exert antibacterial activity through inhibition of DNA gyrase and peptidoglycan synthesis pathways.

### 3.7 MD simulations for stability and flexibility assessment

**FIGURE 14:**
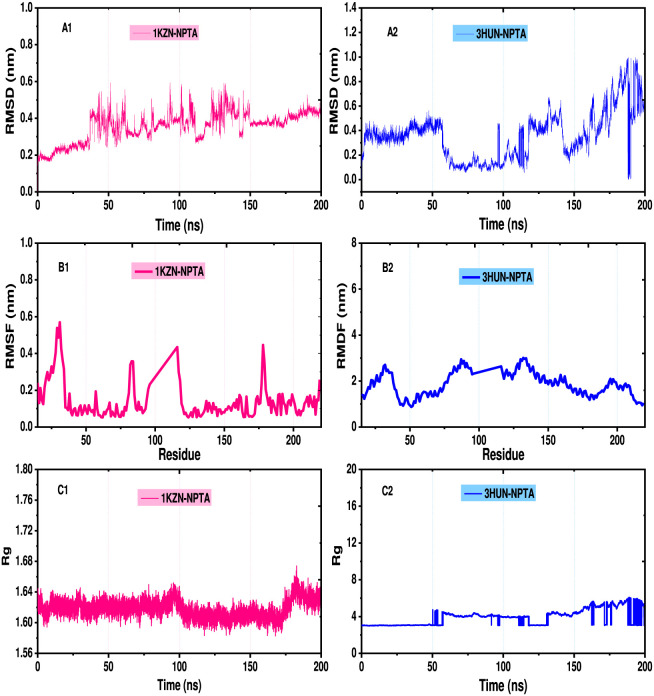
Molecular dynamics simulation of the indicated NPTA-target protein complexes. RMSD **(A)**, RMSF **(B)**, and Rg **(C)** changes during the simulation time. 1KZN, DNA gyrase subunit B; 3HUN, *Staphylococcus aureus* PBP4.

MD simulations were used to evaluate the structural stability, flexibility, and compactness of the NPTA-target protein complexes over time by calculating the RMSD, RMSF, and Rg. The RMSD profiles (Figure 14A) indicated that the 1KZN–NPTA complex exhibited a relatively stable trajectory, with deviations remaining in the range of 0.2–0.5 nm, suggesting that NPTA forms stable interactions such as hydrogen bonds and hydrophobic contacts that restrain conformational changes (Fu et al., 2023). Conversely, the 3HUN–NPTA displayed higher RMSD fluctuations, indicating structural instability and potential perturbations in the ligand.

The RMSF analysis (Figure 14B) provided information on flexibility at the residue level. The 1KZN–NPTA complex had low RMSF values (<0.5 nm) for most residues, indicating restricted mobility and stable ligand binding. Conversely, the 3HUN–NPTA complexe exhibited larger fluctuations (RMSF values >2.0 nm) in some regions, presumably loop regions or flexible binding pockets, indicating lower structural rigidity and potentially weaker protein–ligand retention. These observations are consistent with recent research showing that higher residue flexibility correlates with weaker ligand binding and potential destabilization of the protein–ligand complex (Stachowski and Fischer, 2022).

The Rg plots (Figure 14C) provided information on the general compactness of the protein–ligand complexes during the simulation (Singh et al., 2023). The Rg values of the 1KZN–NPTA complex remained stable, indicating a compact, well-folded structure throughout the simulation. Conversely, the 3HUN–NPTA complex exhibited a consistent increase in Rg, indicating partial unfolding or expansion of domains.

Collectively, these MD simulation results confirmed that the 1KZN–NPTA complex is the most stable (low RMSD, less residue flexibility, and retained compactness) and therefore represents a promising candidate antibacterial agent. Conversely, the 3HUN–NPTA complexe exhibited dynamic instability, undermining their chances of further development.

## 4 Conclusion

In this study, the heterocyclic compound 5-(3-nitrophenyl)-1,3,4-thiadiazol-2-amine (NPTA) was successfully synthesized through a one-pot cyclocondensation reaction and structurally characterized using FT-IR and ^1^H NMR spectroscopy. Thermal analysis demonstrated that the compound possesses satisfactory thermal stability, with decomposition occurring at approximately 210 °C. Antibacterial evaluation against the reference strains *Acinetobacter baumannii* ATCC 17978, *Listeria monocytogenes* ATCC 19114, and *Klebsiella pneumoniae* ATCC 13883 revealed notable inhibitory activity, indicating that NPTA exhibits antibacterial potential against both Gram-positive and Gram-negative bacteria.

Density functional theory (DFT) calculations revealed favorable electronic properties, including a relatively small HOMO–LUMO energy gap, suggesting enhanced chemical reactivity and the potential for intermolecular interactions. The molecular electrostatic potential analysis further identified electron-rich and electron-deficient regions that may contribute to the interaction of NPTA with biological macromolecules.

Molecular docking studies showed that NPTA can be accommodated within the active sites of DNA gyrase subunit B (1KZN) and penicillin-binding protein 4 (3HUN), exhibiting binding energies of −6.8 and −7.0 kcal/mol, respectively. Comparative analysis with the co-crystallized ligands chlorobiocin (−8.83 kcal/mol) and ampicillin (−7.78 kcal/mol), as well as with the reference antibiotics gentamicin and cefazolin, provided a clearer context for interpreting the docking results and suggested that NPTA is capable of establishing favorable protein–ligand interactions. However, these computational findings should be considered supportive rather than definitive evidence of the compound’s mechanism of action.

Molecular dynamics simulations further demonstrated the stability of the 1KZN–NPTA complex throughout the simulation period, whereas the 3HUN–NPTA complex exhibited comparatively greater structural fluctuations. These observations support the potential of NPTA to maintain stable interactions with selected antibacterial targets under dynamic conditions.

The combined experimental and computational findings identify NPTA as a biologically active thiadiazole derivative with encouraging antibacterial properties. Nevertheless, since the biological evaluation was performed exclusively on reference strains, additional studies involving resistant clinical isolates, target-based biochemical assays, and *in vivo* investigations will be necessary to further elucidate its mechanism of action and assess its potential for future antibacterial development.

## Data Availability

The original contributions presented in the study are included in the article/supplementary material, further inquiries can be directed to the corresponding authors.
